# Perceptions of Preventive Health Care and Healthy Lifestyle Choices for Low Income Families: A Qualitative Study

**DOI:** 10.5402/2013/189180

**Published:** 2013-04-03

**Authors:** Sasha A. Fleary, Reynolette Ettienne-Gittens, Robert W. Heffer

**Affiliations:** ^1^Texas A&M University, Department of Psychology, MS 4235, College Station, TX 77843, USA; ^2^University of Hawai'i Cancer Center, Epidemiology Program, Honolulu, HI 96813, USA

## Abstract

This paper examines Head Start parents' perceptions of preventive health and healthy lifestyle choices and Head Start administrators' perceptions of the needs of parents they serve. To address the preventive health of the population, it is necessary that we explore perceptions, risks, and protective factors of preventive health. Focus groups were conducted with parents and administrators to elicit this information and to obtain suggestions for improving preventive health and healthy lifestyle choices among this group. Overall, nutrition and physical activity emerged as themes in parents' definition of preventive health and healthy lifestyle choices. They further identified social support and education as major protective factors for engaging in preventive health and healthy lifestyle choices. Results of this study can be used to inform research and practice to develop interventions to increase preventive health and healthy lifestyle choices among low income families.

## 1. Introduction

Low income persons in the USA are less likely to seek preventive health care and have low health literacy, which places them at risk for poorer health outcomes and increased use of treatment services [1–3]. Health literacy may impact on individual's ability to successfully use the health care system and to engage in preventive care and disease management [4]. In the case of parents of young children, low health literacy may compromise their ability to seek or engage in preventive care for themselves and their children. According to the Institute of Medicine [1], older adults, racial and ethnic minorities, individuals with less than a 12th grade education, GED certificate recipients, nonnative English speakers, and individuals with low incomes are all more likely to have low health literacy. Individuals with these demographic characteristics are also more likely to be socially disadvantaged and thus further disadvantaged on health.

Although a plethora of research exists on the effects of health literacy on adult health, relatively less research has been conducted to address the impact of parental health literacy on the health and well-being of children. Children, especially preschool-aged children, are vulnerable to their environment [5] and rely heavily on their parents to ensure their health and well-being. Based on the adult studies, therefore, parents' health literacy is postulated to influence the preventive health measures parents pursue for their children. 

## 2. The Head Start Program 

The Head Start Program was established in 1964 as part of the “war on poverty.” This program serves low income families and is a federally funded initiative designed to promote school readiness by the enhancement of the social and cognitive development of children through educational, health, nutrition, social, and other services to them and their families. The program, which has helped over 30 million children since its inception, serves newborns and children up to age five [6]. During the 2007-2008 period, approximately 52% of the Head Start families receiving services nationally were ethnic minorities (excluding “unspecified”), 32% of parents had less than a 12th grade education, and for 30% of them, English was not the primary language spoken at home [7]. Early Head Start serves infants, toddlers, pregnant women, and their families. Head Start recipients have incomes below the federal poverty level. Prior research has illustrated that Head Start parents may have lower health literacy, be socially disadvantaged, and make fewer healthy lifestyle choices for themselves and their children. For example, Hudson et al. [8] conducted a study among Head Start parents and children and found that almost all the children ate less than the recommended serving of fruits and vegetables per day and that higher snack consumption was related to less active play per week. They also found that parents perceived their children to be at a healthier weight than was factual. 

 We sought to examine Head Start parents' and administrators' risk and protective factors and views on preventive health and achieving a healthy lifestyle. In so doing, we hope to facilitate the assessment and response to the health literacy and preventive health needs of this population to promote and improve preventive health and healthy lifestyle choices. 

 Implications of this paper should facilitate development of interventions to address Healthy People 2020 [9] preventive health goals and related objectives.

## 3. Method

### 3.1. Participants, Procedures, and Study Design

Three focus groups were conducted in 2010 to determine Head Start administrators' (HSAs) and parents' perceptions of preventive health and healthy lifestyle choices. Two focus groups were conducted with parents (one was conducted in Spanish) and one focus group was conducted with HSA. Head Start parents were recruited by administrators via fliers and direct personal communication. The local Head Start director was instrumental in recruiting HSA. There were both men and women in the parent sample. The HSA sample comprised of 6 persons. The aims of the focus groups included (1) to determine how low income persons defined a healthy lifestyle and preventive health; (2) To determine the barriers and protective factors for seeking preventive health and healthy lifestyle choices; (3) to obtain insight from parents and administrators on what could be done to improve preventive health and healthy lifestyle choices among parents.

HSA and English-speaking parents' (EFG) focus groups were moderated by one of the authors. The Spanish-speaking parents' focus group (SFG) was conducted in Spanish and was coled by two trained moderators, including an HSA. All participants provided their informed consent prior to the focus group questions being administered. Semistructured discussion questions were developed *a priori* and used to guide the focus groups. 

Questions presented to HSA were designed as a *needs assessment* to ascertain the perceived needs of parents from the perspective of the administrators. Focus groups lasted approximately 90 minutes. Each focus group meeting was audio-recorded. All study procedures were approved by the Institutional Review Board of Texas A&M University and by the local Head Start Policy Council. Participants completed a demographic questionnaire before the focus group began and were compensated with a $30 gift card at the end of the group. Dinner and childcare were provided. The groups were conducted in a community location that was accessible and familiar to families.

### 3.2. Data Analysis

Focus group recordings were transcribed verbatim. The SFG was transcribed and translated by a native Spanish speaker. A second Spanish speaker transcribed and retranslated the original transcription, and transcripts were checked for consistency in translation. Handwritten notes were also obtained during the focus group sessions. These notes served to enhance the audio of the interviews. After the groups were transcribed, all transcripts were read in their entirety several times. Coding then occurred, transforming and combining the raw data into units, which allowed for the description of relevant content characteristics [10]. These units were then content analyzed separately by two of the authors and themes were established.

## 4. Results 

A total of 18 parents participated in the focus groups. The majority of the parents were Hispanic (56%) with 90% of the participants in this group indicating that they spoke primarily Spanish at home, a proxy indicator of their level of acculturation. These results can be found in Table 1. The mean age of the parents was 28.5 ± 12.84 (EFG) and 30.13 ± 4.91 (SFG), respectively. The majority of parents indicated that they did not work, with the majority of both groups of parents describing their health as good or excellent. More than half of the participants in both samples participated in the WIC program, a federal supplemental program which provides nutrition information and low-cost nutritious foods to low-income pregnant and postpartum women and their children. Additional demographic information on both samples of parents is presented in Table 1.

 The HSAs were all female and primarily nonHispanic white (67%). On average they had been serving Head Start families for more than six years, with one participant employed at Head Start for more than 18 years (results not presented in Table 1). 

 From the qualitative analysis, several central themes emerged. 

### 4.1. Preventive Health

#### 4.1.1. Definition

 Both groups of parents expressed the view that preventive health encompassed some aspect of nutrition and exercise. This included educating themselves and their children on the recommendations for and the benefits of regular exercise and eating well. Parents also mentioned concerns related to immunizations and regular physician checkups. One parent in the EFG likened preventive health to maintaining a vehicle;

*…it's like a car you know, take it to the shop your car, making sure its oiled and maintenance you know, make sure it's running right and tuned, that's what I try to do with my child,… make sure its tuned up for allergies and stuff like that…*

There were also noteworthy differences between the EFG and SFG. Parents in the EFG tended to highlight treatment of illnesses, while the SFG highlighted prevention “it's prevention before having any type of illnesses and to help us, well yes, not develop the symptoms from said illness…that is like prevention before preventing.” Additionally, the EFG did not mention sleep in their description of preventive health, whereas the SFG identified sleep as being a part of preventive health and made the connection between sleep and illness. Conversely, the EFG discussed social health as being an important aspect of preventive health “social health is also important as well as the physical health…they walk hand in hand to health even for adults.” Other representative quotes from parents can be found in Table 2. 

#### 4.1.2. Preventive Health Protective Factors—Parents

 Parents reported that knowing the benefits of preventive health made them more likely to engage in preventive health. Additionally, they indicated that if they were provided with guidelines of where and when they could seek care for their children as well as received more information regarding proper nutrition, reading food labels, as well as hygiene practices to teach their children, it would serve to reduce the barriers they faced in seeking preventive health. They also identified social support as an important protective factor. Quotes provided by parents are outlined in Table 2. 

#### 4.1.3. Preventive Health Protective Factors—Head Start Administrators

Based on their many years working with families, HSA agreed that providing “one stop shopping” for health care services increases the likelihood that their families receive preventive health services for their children. “One stop shopping” involves having all the providers in one place at the same time. They explained that this minimized transportation issues and the amount of time parents would require away from their jobs. HSA also believe that providing transportation increased compliance with preventive health services. They reported that encouragement and support from staff in addition to helping families navigate their insurance coverage and families' comfort with providers make it easier for families to seek preventive services. HSA identified that educating children and using them as models at home for preventive health also encourages preventive health. 

#### 4.1.4. Preventive Health Barriers—Parents

Both EFG and SFG identified themes related to access to the medical world, including lack of transportation to attend visits and lack of insurance. They expressed difficulties with navigating the insurance system as well as having limited access to medical professionals to express and discuss concerns as well as ask questions. The EFG identified the inflexible schedules of doctors as a barrier to them seeking preventive health for them and their children. Both groups identified the language of printed materials they receive at the doctors' offices as barriers. For the EFG, the terminology used and readability of the information prevented them from fully understanding the material. For the SFG, the translation of the materials posed a problem, with one parent indicating, “The problem is, do you know what the problem is, sometimes they do not translate it correctly…if they give it to me in Spanish, give it to me also in English because I can read in English a little bit. There are some words that I do not understand.”

#### 4.1.5. Preventive Health Barriers—Head Start Administrators

HSA identified finances, transportation, and time as major barriers to preventive health for families they serve. They also reported that their families had trouble accessing health care because of the high prevalence of insurance lapses. HSA agreed that families' lack of knowledge was often responsible for insurance lapse. Lack of knowledge also affected the type of care families sought (treatment versus prevention). HSA further identified language, culture, religion, and immigration as other potential barriers to preventive health for their families.

### 4.2. Healthy Lifestyle Choices

#### 4.2.1. Definition

When asked their definition of healthy lifestyle choices, both EFG and SFG identified common themes of living healthy, including avoiding smoking and drinking, nutrition, and exercise. The SFG mentioned the importance of engaging their children in healthy lifestyles especially as related to exercising as a family and modeling healthy eating behaviors. The same was echoed in the EFG, as one mother provided the following narrative:

*The way we live healthy, like what you eat, how you exercise, or keep yourself physically,…but you have to also like keep physically fit, you cannot just be like just because you walk or move around, you have to be physically fit, your body has to be like, you know just being….*

Similar to their definitions of preventive health, the EFG included social health including quality time with their family in their definition of healthy lifestyle choices, while the SFG focused on physical health. The EFG also included seeking information about health in their definition of healthy lifestyle choices.

#### 4.2.2. Healthy Lifestyle Choices Protective Factors—Parents

Both groups of parents identified community and social support as facilitators for them engaging in healthy lifestyle choices. One mother in the SFG believed that parents needed to unite for outings to the park and other community locations, as she was often fearful of going to these places with her three children alone. She commented that when going home after dark with her children, she had to be hyper vigilant, and aware of her surroundings. The EFG expressed the desire for peer support as a method of encouraging them to engage in healthy lifestyle choices. They believed that a support group would serve to hold each other accountable and increase awareness of the need for healthful practices in the community. 

#### 4.2.3. Healthy Lifestyle Choices Barriers—Parents

Both the EFG and SFG indicated that environmental issues pertaining to neighborhood safety (e.g., exposure to potentially harmful individuals such as sex offenders and lack of sidewalks) prevented them from seeking healthy lifestyle choices, particularly exercising outside. Parents were fearful of allowing their children to play outside in parks alone and of being in certain parks with their children at dusk. To address this, they reportedly encouraged their husbands to exercise with the family; however, they experienced barriers here as well, as the men were often either uninterested or too tired to go outside with the rest of the family. Additionally, the SFG identified lack of communication with physicians, acclimating to a different (from homeland) health care system and insurance as barriers to them engaging in healthy lifestyle choices. 

#### 4.2.4. Healthy Lifestyle Choices Barriers —Head Start Administrators

 Similar to preventive health, HSA identified time and finances as major barriers to parents making healthy lifestyle choices. They also identified culture and education as barriers. HSA agreed that a high prevalence of mental health problems and stress exists among the families they serve, and this hinders families' abilities to make healthy lifestyle choices. HSA, however, believed that in addition to environmental barriers that may prevent children from exercising outside, parents did not make engaging in a healthy lifestyle an important priority. 

### 4.3. What Do Families Want and Need?

#### 4.3.1. Education

The EFG and SFG both stressed the need for more education about preventive health and healthy lifestyle choices. Specifically, they wanted education on obesity, oral health, vision health, ear health, nutrition, physical activity, child development (including behavior management), and food allergies. Parents stated that they would also like their children to be educated about preventive health and healthy lifestyle strategies and wanted to be educated on their children's diagnoses and medical issues. Both groups desired education on available community resources with the EFG wanting more awareness via talks and seminars and presented in easy-to-read pamphlets and fliers.

HSA were able to identify several areas of preventive health that they believed the parents would benefit from. These needs were identified as education related to smoking habits, baby bottle syndrome, and vaccinations. They believed that parents would also benefit from education regarding nutrition, physical activity, and sleep. HSA maintained that it would be important to address the relationship between preventive health and school performance, in addition to preventive health and general health for parents. They believed that preventive health education should encompass information regarding the connections between convenience (fast) foods versus home cooking and portion and serving sizes, as related to children's weight status. HSAs also believe that parents would benefit from education in acquiring and keeping health insurance and choosing a primary care medical provider.

#### 4.3.2. Intervention Etiquette

HSAs believe that preventive health interventions needed to be accessible to increase enrollment and retention. Accessibility entails conducting interventions in the community at familiar convenient locations and at convenient hours. They further provided examples of a program which had achieved some success, brought the providers to the parents, “We bring the providers to the school, we bring the providers to the community room, where they can get their physicals, their immunizations and their dental treatment all in the same place…and we transport too.” Reminder calls, providing meals, and childcare as well as reaching out to individual parents aided in parents attending the sessions. 

Regarding interventions aimed at Head start parents, HSA believed that parents they serve gained the most knowledge from experiential learning. They also believe that information should be delivered visually and audibly and if needed the use of bright, colorful, and eye-catching printed materials. 

#### 4.3.3. Policy Change

Participants in all three focus groups identified the red tape associated with Medicaid coverage as a major barrier to preventive health. Participants agreed that clearer guidelines for acquiring and maintaining Medicaid coverage would increase the amount of children who are ensured, reduce the number of children with a lapse in coverage, and improve compliance with preventive health doctors' visits. 

## 5. Discussion

This study provides a rich source of information from the perspective of low income persons on two areas of research, preventive health and healthy lifestyle choices that are pertinent and timely. Although low income parents in Texas have some idea of what is needed to keep themselves and their children healthy, they experience financial, institutional, and environmental barriers while trying to do so. Recent research on the health of Americans confirms that socioeconomic status (poverty, employment, and educational attainment) affects one's ability to access preventive health services [11]. 

In concordance with other research conducted with Head Start participants [12], the parents in our sample were aware of the benefits of exercise, modeling healthy behaviors, as well as providing healthy foods for their children. However, parents mentioned that lack of time and energy posed a barrier to really achieving this, similar to results found by other researchers [12–14]. The EFG and SFG shared several similar perceptions, needs, risks, and protective factors. However, some of their perceptions and concerns were qualitatively different (e.g., language of printed materials at doctors' offices), and some were unique to a specific group (SFG inclusion of sleep in their perceptions of preventive health). 

This study has several limitations. First the findings were from a small sample of Head Start parents. It is possible that these parents who volunteered for this research were highly motivated and informed and as such may not be representative of all Head Start parents in the sampled community. The generalizability of our findings therefore is not known, given the selective group utilized for the research and the geographical limitations posed by residing in a college town in Texas as well as the southern region of the USA.

These focus groups provided a plethora of information that can inform research and practice, some of which are highlighted in the “What do families want and need” section of this paper. Interventions to increase preventive health and healthy lifestyle choices should incorporate both risk and protective factors of this selected population to maximize success. 

## Figures and Tables

**Table 1:** Characteristics of study participants for the parent focus groups.

	English-speaking parents *n* = 8	Spanish-speaking parents *n* = 10
	*n* (%)^a^	
Sex		
Men	1 (12.5)	1 (10)
Women	7 (87.5)	8 (80)
Race/ethnicity		
Non-Hispanic white	3 (37.5)	—
Non-Hispanic black	3 (37.5)	—
Hispanic/Latino	2 (25)	10 (100)
Language spoken at home		
English	6 (75)	—
Spanish	1 (12.5)	9 (90)
Both English and Spanish	—	1 (10)
Employment status		
Full time	1 (12.5)	2 (20)
Part time	1 (12.5)	—
I do not work	6 (75)	7 (70)
Participation in the WIC^b^ program		
No	2 (25)	4 (40)
Yes	6 (75)	6 (60)
Education		
≤12th grade	—	4 (40)
HS Grad/GED	2 (25)	2 (20)
≥Some college	6 (75)	1 (10)
Total yearly household income		
≤10,000	3 (37.5)	3 (30)
10,001–20,000	1 (12.5)	5 (50)
20,001–30,000	3 (37.5)	2 (20)
I do not know	1 (12.5)	—
Perceived health status		
Excellent	3 (37.5)	3 (30)
Good	4 (50)	6 (60)
Fair	1 (12.5)	1 (10)
Poor	—	—

^a^Percentages do not add up to 100 due to missing data.

^b^The special supplemental nutrition program for women, infants, and children (WIC).

**Table 2:** Main focus group themes and representative quotes.

Concept	Theme	Representative quotes
Defining preventive health	Definitions varied by group; however parents both identified nutrition and physical activity (exercise) as encompassed in preventive health.	“physical exercise and diet,” “Regular dental checkups,” “social health is also important as well as the physical health,” “its prevention before having any type of illnesses and to help us, well yes, to not develop the symptoms from said illness”

Barriers to preventive health	Navigating the medical world	“Insurance is really important,” “sometimes doctor's office might be booked and they don't have and they don't have openings and that might be the only time the patient will be able to come in to be seen,”
	Cultural barriers	“that sometimes we don't know because of the upbringing they taught us in Mexico. It is very different here,” “We tend to think that the chubby children are the healthiest children… and we say that the chubbier the child is the healthier he is and that is not true. Because sometimes when we have family reunions, sometimes I feel embarrassed that my daughter is not that chubby.”

Protective factors to preventive health	Knowledge of benefits of preventive health	“We can get more information regarding nutrition, read the nutrition labels that have everything, have that information..”
	Guidelines for preventive health	“Like providing guidelines, providing guidelines of where to do checkups you know or you know, what to seek medical attention on,” “And having seminars or you know talks, like -I like what they do it here at [Center] and of course it's in conjunction with head start…”

Defining healthy lifestyle choices	Living healthy	“like what you eat, how you exercise, Or keep yourself physically,” “Its just like making choices you know get outta the house and take your kid to the park, even though you're, yea, you're tired and you work all day long,” “Because a healthy lifestyles should have everything, exercise, nutrition and hygiene, a good rest.”

Barriers to healthy lifestyle choices	Neighborhood safety	“and neighborhoods like poor low income neighborhoods, sometimes they don't have a safe environment to be walking around,” “so there's not sidewalks for them to walk on”
	Lack of communication with physicians	“Sometimes the doctors don't diagnose,” “But my doctor would tell me that everything was okay,” “security is the top priority for this for healthy lifestyle -security would be first at the top of the list”

Protective factors to healthy lifestyle choices	Community and social support	“sometimes you to need someone to encourage you,” “that's when encouragement comes in because sometimes you to need someone to encourage you hey you say you wouldn't go to on a walk by yourself hey lets how about we go for walk”
